# Taming transposable elements in livestock and poultry: a review of their roles and applications

**DOI:** 10.1186/s12711-023-00821-2

**Published:** 2023-07-21

**Authors:** Pengju Zhao, Chen Peng, Lingzhao Fang, Zhengguang Wang, George E. Liu

**Affiliations:** 1 Hainan Institute of Zhejiang University, Hainan Sanya, 572000 China; 2 College of Animal Sciences, Zhejiang University, Zhejiang Hangzhou, People’s Republic of China; 3 Center for Quantitative Genetics and Genomics, Aarhus University, 8000 Aarhus, Denmark; 4 Animal Genomics and Improvement Laboratory, Beltsville Agricultural Research Center, Agricultural Research Service, USDA, Beltsville, MD 20705 USA

## Abstract

Livestock and poultry play a significant role in human nutrition by converting agricultural by-products into high-quality proteins. To meet the growing demand for safe animal protein, genetic improvement of livestock must be done sustainably while minimizing negative environmental impacts. Transposable elements (TE) are important components of livestock and poultry genomes, contributing to their genetic diversity, chromatin states, gene regulatory networks, and complex traits of economic value. However, compared to other species, research on TE in livestock and poultry is still in its early stages. In this review, we analyze 72 studies published in the past 20 years, summarize the TE composition in livestock and poultry genomes, and focus on their potential roles in functional genomics. We also discuss bioinformatic tools and strategies for integrating multi-omics data with TE, and explore future directions, feasibility, and challenges of TE research in livestock and poultry. In addition, we suggest strategies to apply TE in basic biological research and animal breeding. Our goal is to provide a new perspective on the importance of TE in livestock and poultry genomes.

## Background

Livestock and poultry play a crucial role in human survival and development. They are capable of converting low-quality feed into high-quality protein and essential minerals with high bioavailability, which can be easily incorporated into human diets. Currently, a significant amount of research on livestock and poultry focuses on genetic resources, cis-regulatory elements, gene regulatory networks, and epigenetics [1–5]. A comprehensive understanding of the genomic structure is especially important, as it lays the foundation for investigating important economic traits in livestock and poultry using biological approaches and mechanisms.

Compared to well-studied single nucleotide polymorphisms (SNPs), TE are mobile, repetitive, and diverse genomic elements that occupy a larger portion of eukaryotic genomes [6]. Transposable elements were initially viewed as “selfish” DNA or “parasitic” elements because of their deleterious effects on host genomes [7]. However, recent studies have demonstrated that TE play important roles in driving the evolution of genomes [8]. Transposable elements can promote genetic diversity through insertion [9] and regulate other factors such as genome size expansion [10], 3D organization [11], chromatin modifications [12], gene regulatory networks [13], and DNA methylation [14]. Transposable elements can be considered as a source of raw material for primitive genomes, tools of genetic innovation, and ancestors of modern genes (e.g., ncRNA) [15]. Transposable elements are able to affect conserved and divergent chromatin looping and contribute to cell- and species-specific gene regulation [11]. Moreover, TE can be regulated by context-specific patterns of chromatin marks in embryonic stem cells [16], and TE-driven DNA methylation allows genome expansion [17].

In spite of the abundance of research on the roles of TE on the genome biology in humans, model organisms (e.g., mice and *Drosophila*), and plants (especially crop species), few studies on TE have been conducted in livestock and poultry. Since 2000, there are only 72 studies on TE in livestock and poultry genomes, compared to nearly 1700 studies in humans (PubMed database). Nearly 60,000 polymorphic TE have been found in humans. Some of them are related to expression quantitative trait loci (eQTL) and genome-wide association studies (GWAS) [18]. In plants, some researchers have successfully used TE to improve the economic properties and stress resistance of crops. For example, at least 40 TE insertion polymorphisms have been found to be robustly associated with extreme variations in the major agronomic traits of tomatoes. In addition, a Copia long terminal repeat (LTR)-retrotransposon insertion was reported to be associated with high levels of 2-phenylethanol, which gives a pleasant flowery aroma to tomatoes [19]. In maize, a miniature inverted-repeat transposable element (MITE) inserted into the promoter of the *NAC* gene (*ZmNAC111*) has been found to enhance drought tolerance at the seedling stage [20]. In rice, the insertion of an LTR-retrotransposon into the promoter of the *OsFRDL4* gene (*Os01g0919100*) was reported to enhance its expression level and promote tolerance to aluminum toxicity [21].

The genomes of livestock and poultry contain active and functional TE. For example, the insertion of short interspersed nuclear elements (SINE) into the intron of the porcine *growth hormone receptor* (*GHR*) gene can reduce its expression by acting as a repressor [22]. Moreover, the insertion of a long interspersed nuclear element (LINE) into the 5′UTR of the *agouti signaling protein* (*ASIP*) gene promotes a nearly 10-fold increase in its expression and leads to white coat color in buffalo [23]. However, there is a general lack of a comprehensive understanding of TE in livestock and poultry, and researchers have limited knowledge regarding the bioinformatics strategies and methods of analyzing TE. Therefore, there has been little research on associating TE with economic traits in livestock and poultry.

In this review, we highlight the roles and potential applications of TE in livestock and poultry research as below: (1) we provide an integrated perspective on TE composition and polymorphism in 16 livestock and poultry species; (2) we summarize the potential roles of TE in livestock and poultry species in the past 20 years and discuss the shortcomings of current research, (3) we provide bioinformatic strategies for analyzing TE and list resources suitable for the application of TE in livestock and poultry species, and (4) we discuss ideas and prospects related to the applications of TE in biological research and animal breeding.

### Mobile genetic elements in livestock and poultry

**Fig. 1:**
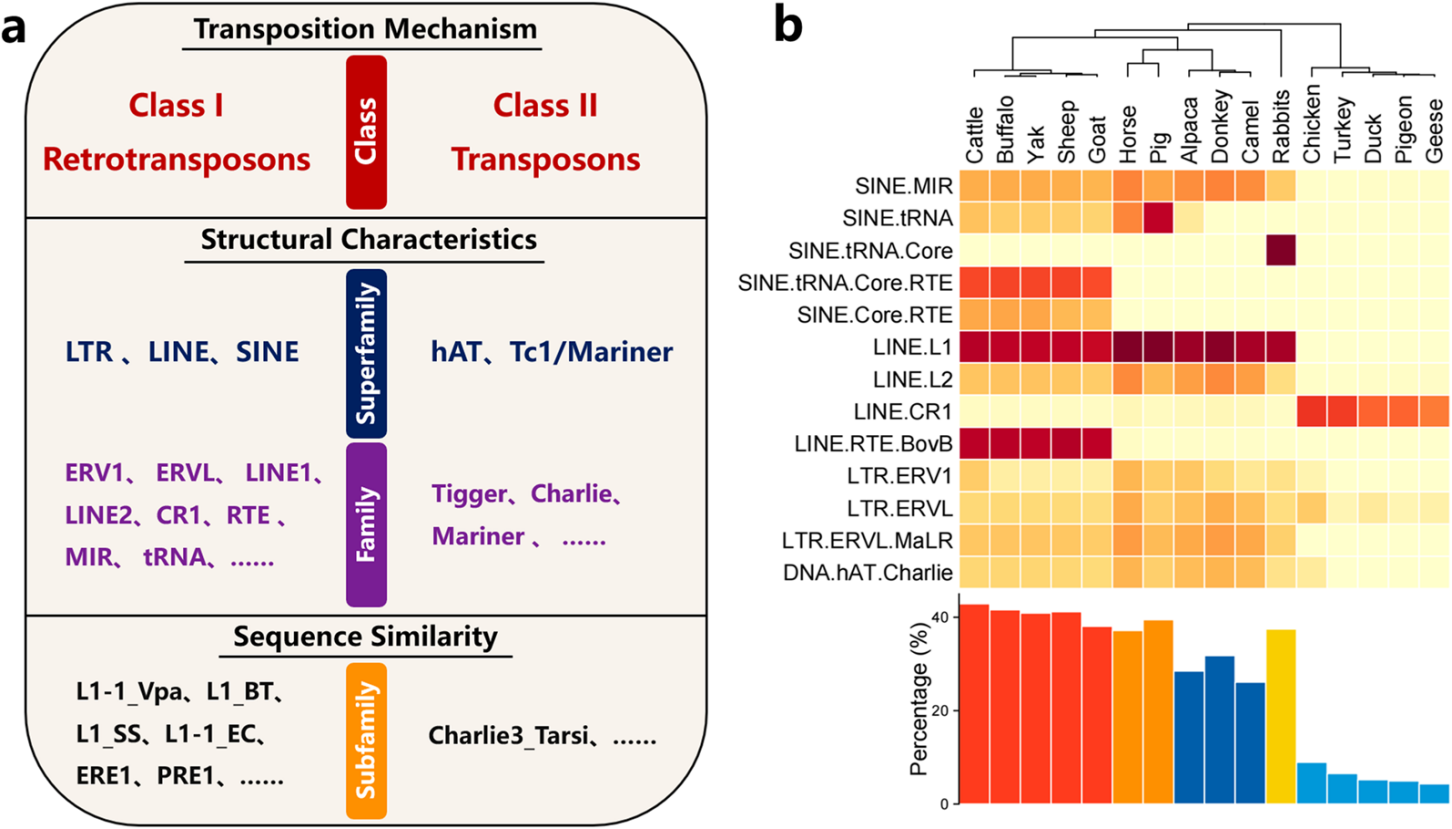
Transposable elements classification and annotation in the livestock and poultry genome: **a** main TE types and classification basis for TE classes, superfamilies, families, and subfamilies; and **b** genomic TE content and genome coverage of representative genomes in 16 livestock and poultry species. The cladogram of the species is based on the clustering of the TE distribution pattern. The heat map shows the level of enrichment, with darker shades of red indicating greater significance

In this section, we summarize the TE that are annotated in 16 livestock and poultry species using species-specific TE libraries retrieved from the Repbase Update database [24] and compare their uniqueness and dynamics (Fig. 1a). Transposable elements can be broadly divided into two classes according to their mechanism of transposition (retrotransposons or transposons). Class I includes LTR and non-LTR retrotransposons (LINE and SINE), and Class II comprises DNA transposons (hAT and Tc1/Mariner) [25]. LINE and SINE typically make up the majority of the mammalian genome and have been shown to be closely associated with genome rearrangements, epigenetic regulation, and human structural variation-related diseases [26]. These classes can be further divided into distinct families and superfamilies based on their DNA sequence, structural characteristics, and phylogenetic analysis.

Our summary of genomic TE content is based on the available representative genomes (retrieved from NCBI) of 16 livestock and poultry species. The TE that we found belong to 13 TE superfamilies, including almost all major TE superfamilies (top 10 in genome coverage) that exist in livestock and poultry (Fig. 1b). The TE landscapes of livestock and poultry genomes showed large differences in abundance and composition. They were dominated by LINE and SINE in terms of genome coverage. In addition to non-LTR elements, LTR elements, although less abundant, are shared across all livestock species and have been shown to be significantly functionalized. In accordance with their size, poultry genomes (genome coverage: 4.3 to 8.9%) have a much lower proportion of TE abundance than livestock genomes (genome coverage: 26.1 to 42.9%). Poultry genomes are mainly dominated by the LINE/chicken repeat 1 (CR1) superfamily, whereas livestock genomes share multiple key TE superfamilies (e.g., LINE/L1). The TE composition shared across Bovidae genomes is unique in many respects compared with those of other livestock species (e.g., LINE/RTE-BovB).

**Fig. 2:**
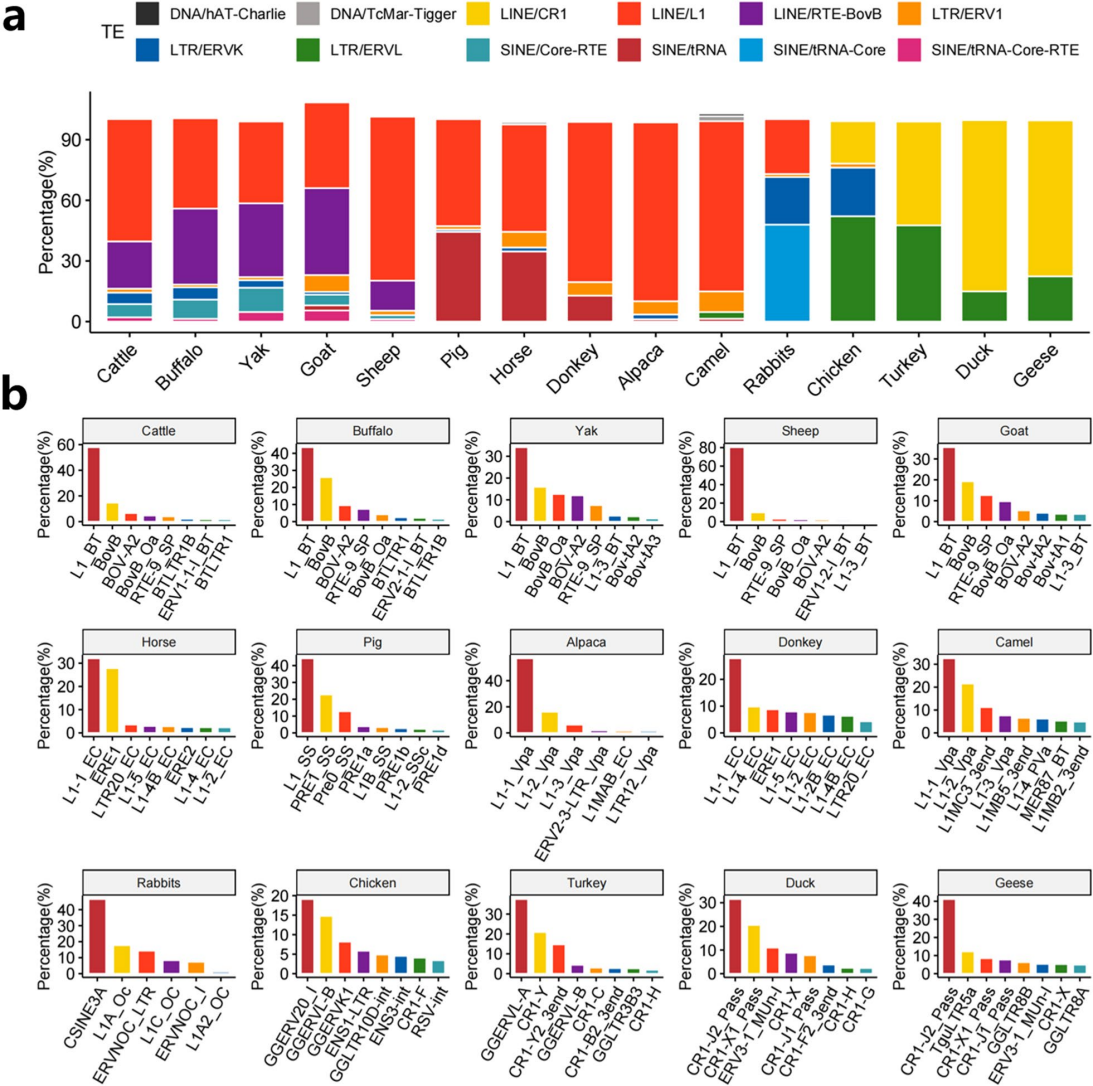
Genomic content of TE superfamilies and families: **a** percentage of different TE superfamilies/families per species; and **b** percentage rankings of various TE subfamilies in 16 livestock and poultry species

Transposable elements contribute highly to the genetic diversity of species, but their contribution to livestock and poultry genomes may have been underestimated in previous studies. Transposable elements with polymorphisms represent the youngest and most active TE, and deserve more attention. The composition and proportions of polymorphic TE superfamilies vary widely among species (Fig. 2a). For example, LINE contribute major genetic polymorphisms to the genomes of livestock and poultry. This is mainly manifested in LINE/L1 in livestock genomes, LINE/CR1 in poultry genomes, and LINE/RTE-BovB in Bovidae genomes. Although LTR/endogenous retrovirus (ERV) group L members (ERVL) have a lower genome coverage relative to LINE/L1 in poultry genomes, ERVL contribute to a large number of polymorphisms. The proportion of the LTR/ERV group K members (ERVK) superfamilies is higher in the chicken genome than in the genomes of other poultry species. Moreover, this LTR superfamily contributes more to the genomic diversity in chickens than the LINE/L1 superfamily, indicating that these ERV have potential biological functions that deserve more attention in future studies on the chicken genome.

The diversity of polymorphic TE families varies widely among organisms. This is true even for shared TE superfamilies, such as LINE/L1 and LINE/CR1. The active mobile elements in most livestock genomes are dominated by one or two types of non-LTR families (Fig. 2b): L1-BT and BovB in Bovidae, L1-1-EC and ERE1 in the horse and donkey, L1-SS and PRE1_SS in Pig, L1-1-Vpa and L1-2-Vpa in the alpaca and camel, and CSINE3A and L1A-Oc in rabbits. The family classes of LINE/CR1 also vary among poultry species, and the mobile elements in these genomes are partly due to the differential amplification of LTR retrotransposons. GGERV elements constitute a major proportion of the polymorphic TE in chicken and turkey, whereas TE in duck and geese are dominated by polymorphic CR1-J2-Pass and CR1-X1-Pass. Targeted research on these active transposons will help elucidate the important role of TE in the functional genomes of livestock and poultry.

### Established knowledge regarding TE in livestock and poultry genomes

**Fig. 3:**
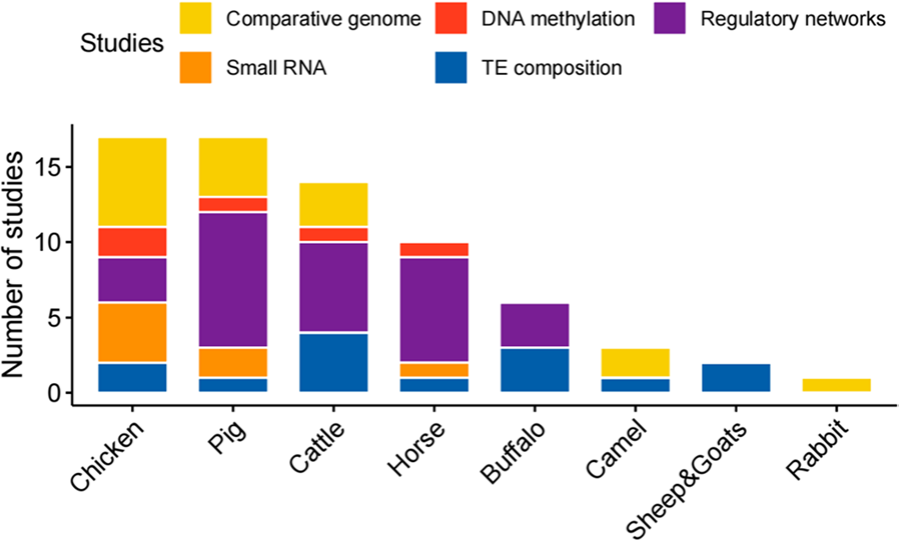
Statistics of 70 studies on 16 species of livestock and poultry TE. There are five major research areas related to TE for the study of livestock and poultry: comparative genomics, DNA methylation, regulatory networks, small RNA, and TE composition

With the emergence of large-scale multi-omics data analysis, studies have gradually revealed the roles of TE in various biological functions in livestock and poultry species. However, these TE have received little attention compared to the TE in humans. In this paper, we reviewed 72 studies on TE in 16 species of livestock and poultry (Fig. 3). These studies mainly focused on TE in three major farm animal species (chicken, pig and cattle) and one companion animal (horse), with little or no research on TE in the remaining species. At the current stage of research in livestock and poultry, the studies have primarily covered investigations of TE composition (21% of the studies) and comparative genomics (24% of the studies). In particular, studies on chickens have involved research on avian evolution and comparative genomics from the perspective of TE. Nearly one-third of the studies are related to gene regulation, and exons, promoters, or intron regions of 13 genes are found to be affected by TE (Table 1). Interestingly, studies on different livestock and poultry species have reported that TE primarily affect genes by altering the first intron region. This may reflect the ascertainment bias introduced by our better understanding of the functions of the promoter regions.

**Table 1:** Summary of the effects of TE on livestock and poultry genes

Species	Superfamily	Gene	Targets	Trait	Ref.
Buffalo	LINE	*ASIP*	First intron	Coat color	[23]
Cattle	LTR	*APOB*	Exon	Cholesterol deficiency	[27]
Cattle	SINE	*TP53*	Promoter	Milk persistency	[28]
Cattle	LTR	*IFNAR2*	Enhancer	IFN immunity	[29]
Cattle	SINE	*IL2RB*	Enhancer	IFN immunity	[29]
Horse	LTR	*TRPM1*	First intron	Complex spotting	[30]
Horse	SINE/LINE	*PYGM*	Exon/intron	Exercise capability	[31]
Horse	SINE/LINE	*BMAL1*	Exon	Circadian rhythm	[32]
Horse	SINE	*MSTN*	Promoter	Speed and stamina	[33]
Pig	SINE	*GHR*	First intron	Skeletal accretion	[22]
Pig	SINE	*PDIA4*	First intron	Litter size	[34]
Pig	SINE	*FSHb*	First intron	Litter size	[35]
Pig	SINE	*VRTN*	First intron	Vertebral number	[36, 37]
Sheep	LINE	*BMP2*	Upstream	Fat tails	[38]

#### Roles of TE in the pig functional genome

The impacts of TE on gene regulation have received more research attention in pigs than in other livestock and poultry, especially through the contributions of Song et al. [39–41]. The first draft genome assembly of pigs provided new insights into TE composition of pig genomes and revealed 87 novel TE families, including five LINE, six SINE, and 76 LTR families. The LINE1 and porcine repetitive element (PRE, a glutamic acid transfer RNA-derived SINE) families are considered to have expanded in the first half of the tertiary period and are still active in the most recent period [42]. With the assembly of an increasing number of genomes, the TE compositions of different pig breeds have been further identified and compared, which has revealed that TE are the main source of large insertions and deletions in these breeds [43, 44].

Some novel TE families have been discovered to be functional. For example, LTR class I ERV element-mediated chimeric transcripts have been identified and characterized in the porcine RefSeq and EST databases [45]. Song et al. reported that most protein-coding genes and long non-coding RNAs (lncRNAs) contain TE retrotransposon insertions. The same research group also showed that young L1 5′UTR and LTR-ERV possess sense and antisense promoter activities and can be expressed in multiple tissues and cell lines [39]. TE-mediated lncRNA are also found in the skeletal muscles of Bama Xiang pigs, and their transcription start sites are remarkably enriched by LINE and SINE [46]. The effects of TE on gene regulation are also reflected in the 3D chromatin structure, chromatin accessibility, histone modification, and transcription factor binding site (TFBS) [47]. It is worth noting that the age of TE is a key factor that affects their activity and tolerance in the pig genome [48].

Gametogenesis and the embryonic stage are important stages for TE activity due to the occurrence of reprogramming, and pigs are no exception to this. Kong et al. [49, 50] have found that the endogenous small interfering RNA pathway provides a sophisticated balance of regulatory mechanisms for TE (e.g., SINE1B and LTR) activity during pig epigenetic reprogramming. Moreover, a large number of TE families were identified in persistently methylated regions during the reprogramming of germ cells in male and female pigs, suggesting the potential role of TE in intergenerational epigenetic inheritance [51].

At present, pigs are the most explored livestock that have TE polymorphisms identified across the whole genome. However, research has been primarily focused on SINE due to their short sequence length, high integrity, and high density. For instance, Song et al. [40] used comparative genomics to identify large-scale structural variations among pig breeds and found that some variations were mediated by SINE insertions. In addition, they selected 30 SINE retrotransposon insertion polymorphism markers to identify the genetic diversity, differentiation, and population structure of seven Chinese miniature pig populations [41]. In a previous study, we successfully used TE polymorphisms on the X chromosome to infer introgression events between Asian and European pigs [52]. We first detected 211,067 polymorphic SINE at the population level using 374 next-generation sequencing (NGS) data. Based on this, we found that TE can clearly recapitulate known patterns of population admixture in pigs [48].

**Fig. 4:**
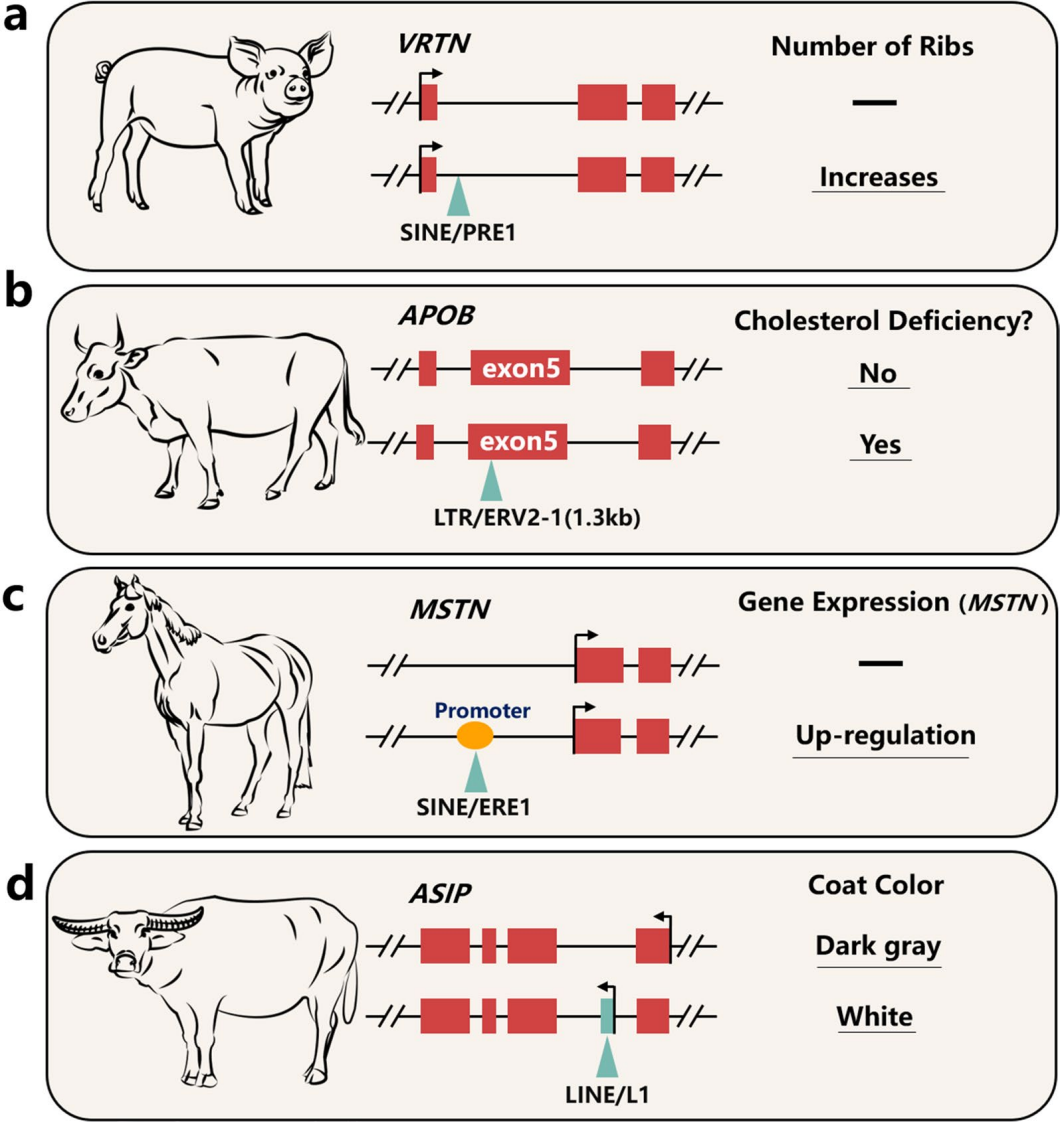
Four examples of the impacts of TE on protein-coding genes: **a** A PRE-1 polymorphism located in the first intron of the *VRTN* gene was found to be significantly associated with the number of thoracic vertebrae in pigs; **b** a 1.3-kb LTR-mediated deleterious mutation in exon 5 of the *APOB* gene was found to cause cholesterol deficiency in Holstein cattle; **c** a SINE polymorphism was found in the promoter region of the *MSTN* gene in thoroughbred horses, which can affect the expression of this gene and **d** the *ASIP* gene in white buffalo lacks pigment in the skin and hair due to a 165-bp insertion of the LINE-1 into its first intron. Red boxes represent exons, green triangles represent TE, and yellow ovals represent promoter

Currently, four genes associated with economically important traits have been found to be similarly affected by SINE in pigs. Of these, the most well-known is PRE-1 in the first intron of *vertnin* (*VRTN*) gene, which is significantly associated with the number of thoracic vertebrae [36, 37] (Fig. 4a). The *follicle stimulating hormone subunit beta* (*FSHb*) and *protein disulfide isomerase family a member 4* (*PDIA4*) genes that are related to the litter size, also have a SINE insertion in their first intron [34, 35]. Moreover, a polymorphic SINE insertion in the first intron of *GHR* serves as a candidate regulator of *GHR* expression by acting as a repressor [22]. These findings help elucidate the role and mechanism of TE in altering genetic variation, as well as their indirect effects on swine phenotypes.

#### Roles of TE in the chicken functional genome

The chicken is an important model organism for studying avian genome structure, function, and evolution. Accordingly, research on TE in the chicken genome has mainly focused on avian genome evolution and epigenetics. Unlike the livestock genome, only approximately 10% of the chicken genome contains TE, which may be the main reason for the small size of the chicken genome [53]. LINE and ERV comprise a major proportion of the TE landscape, and DNA and SINE families exhibit very low activity during the evolutionary history of avian genomes [54–57]. Notably, the chicken repeat 1 (CR1; LINE) retrotransposon is the most active and currently attracts more attention in avian TE research [58]. In fact, CR1 remains active for a long period of time in most orders of neognaths. Its activity level varies significantly between and within avian orders, contributing to lineage-specific changes in genome structure [59]. The CR1 element has been successfully used to clarify the relationships between closely-related galliform species whose radiation and speciation have occurred very recently, indicating that the CR1-based methodology can be used as a powerful tool for phylogenetic research [60, 61]. In addition, there is a small body of research that discusses the functionality of LTR and ERV in chickens; for example, the breed-specific GGERV10B (ERV) insertion site can be used as a specific marker for Korean chickens [62, 63].

The epigenetic silencing of TE is another major component of functional genomics in chickens, and DNA methylation is a key epigenetic mechanism in TE stabilization. Studies have found that changes in DNA methylation in the chicken genome can indirectly affect embryonic muscle development and the body’s immunity to viruses through TE activity [64, 65]. However, unlike the silencing function of the dicer-mediated RNA interference pathway for human L1 retrotransposons, the PIWI-interacting RNA pathway is a key silencing factor for CR1 element repression in chickens [66–68]. Moreover, this pathway exhibits stage-dependent changes in modulating TE for male germ cell development [69].

#### Roles of TE in the cattle functional genome

The cattle genome contains typical eutherian mammalian repeats (e.g., LINE1, MIR, and ERV), and some studies suggest that several BovB (LINE/RTE) elements have been transferred horizontally from Squamata [70, 71]. Both L1_BT and BovB elements have high (~ 10%) coverage in the bovine genome; however, L1 is a younger repeat family than the BovB elements and is likely more active [72].

TE polymorphisms are a major focus of studies on the cattle genome. However, unlike studies on pigs, studies on cattle genomes focus mainly on the detection of low-density transposons at the experimental level. For example, the L1_BT sequence is used as a primer in polymerase chain reactions (PCR) for multi-site genotyping, and is a convenient marker for genetic differentiation between breeds [73]. The Heligloria family of DNA transposons was genotyped using the ISSR-PCR-like method to study the co-localization of DNA transposons (Helitron) and retrotransposons in the genomes of three cattle breeds [74]. Han et al. used NGS data and the droplet digital PCR platform to quantitatively detect Hanwoo-specific structural variations (SV) generated by TE-associated deletion events, and then used these TE to distinguish different cattle breeds (e.g., Hanwoo vs. Holstein) [75, 76].

There are significant differences in the frequency of LINE and SINE in the 100-kb upstream region of female- and male-imprinted genes in cattle [77]. Bov-A2 (SINE) was found to be inserted into the promoter region of the *tumor protein P53* (*TP53*) gene in Antilopinae and Tragelaphini (bovine subfamily and tribe, respectively), but was absent in the *TP53* promoter of the domestic cow and buffalo genomes. This discrepancy may help explain the genetic networks that regulate mammary involution (e.g., cow milk persistency) and lead to phenotypic differences across Bovidae [28]. Importantly, genes related to the type II interferon (*IFN*) response in bovine cells have TE-derived enhancers [e.g., *interferon-alpha/beta receptor beta chain* (*IFNAR2*) and *interleukin 2 receptor subunit beta* (*IL2RB*)], and the corresponding TE are polymorphic in modern cattle [29]. In addition, a 1.3-kb LTR-mediated (ERV2-1) deleterious mutation was detected in the coding region of the *apolipoprotein b* (*APOB*) gene (Fig. 4b). This mutation causes transcripts to be truncated and abnormally spliced, leading to cholesterol deficiency in Holstein cattle. These findings indicate that TE contribute to gene regulation and evolution and play important roles in maintaining immunity in cattle [27].

#### Roles of TE in the horse functional genome

Similar to the cattle genome, the horse genome also has a large number of hybrid repetitive sequences in addition to the typical repetitive sequences of eutherian mammals. In particular, the *Equus caballus* clade-specific LINE 1 (L1) repetitive sequence can be classified into five subfamilies, three of which have undergone recent rapid expansion [78]. In total, 1310 TE were reported to have been integrated into horse mRNA genes, and a small proportion of them have been exonized into coding sequences. The TE inserted into the coding sequence show a preference for antisense orientation, approximately 40% of which are represented by LINE [79]. This feature is also supported by findings from the exercise transcriptomes of equine athletes, indicating that antisense transcription may be one of the main mechanisms of TE regulation in horses under stress conditions [80]. One family of ERV elements (LTR) accounts for the highest proportion of TE insertions into horse coding sequences, and is known to be a donor for miRNA production [81]. They can induce congenital quiescent night blindness and complex spots in horses by affecting the *transient receptor potential cation channel subfamily M member 1* (*TRPM1*) gene [30].

Exercise-related phenotypic characteristics are the most important aspect of the horse functional genome, and TE have been found to play an important role in this regard. For example, LINE-derived sequences are highly and differentially expressed during physical activity by horses [82]. LINE show a high abundance of differentially-methylated regions in the pre- and post-exercise blood samples of superior and inferior horses [83]. In particular, three TE-mediated genes have been found to be related to the athletic ability of horses. The *basic helix-loop-helix ARNT like 1* (*BMAL1*) gene is a key regulator of the circadian rhythm, and its first exon undergoes horse-specific exonization of CR1 (LINE) and MIR (SINE) [32]. The *glycogen* *phosphorylase* *muscle associated* (*PYGM*) gene is involved in providing energy for the body by disassembling glycogen in the muscles, and is highly conserved in mammalian genomes. A study reported TE insertions in the exons and introns of this gene, including an L2 (LINE) exonization event in exon 15 [31]. The *myostatin* (*MSTN*) gene is a significant inhibitor of skeletal muscle growth, and has been shown to account for gene-based race distance aptitude in racehorses. A SINE polymorphism was found in the promoter of this “speed gene” in thoroughbred horses (Fig. 4c). This TE is specifically responsible for adversely affecting transcription initiation and gene expression, thereby limiting the production of the MSTN protein [33].

#### Roles of TE in the functional genomes of other animals

In addition to the four species above, research on TE in other livestock and poultry species—including goat [84], sheep [38], rabbit [85], buffalo [86], and camel [87, 88]—mainly involves the composition and evolution of TE. There may be fewer functional genome and epigenetic annotations available for these species compared to the previously mentioned ones. Undoubtedly, there are probably many functional elements and gene regulation events mediated by TE beyond those that have been reported. These all offer future prospects for understanding species evolution and biological functions from the perspective of TE.

It is worth noting that the conservation of TE insertions is crucial for understanding the impact of TE on functional roles among livestock and poultry. In a previous study, we discovered the insertion of a full-length PRE0-SS (sus-specific SINE) into the 3′UTR of the porcine *pyruvate dehydrogenase kinase 1* (*PDK1*) gene. This was consistent with a previous report showing that Alu and B1 (primate-specific and rodent-specific SINE, respectively) regulate the human and mouse orthologs of *PDK1* through Staufen-mediated decay, respectively [89]. In addition, we previously reported that the 165-bp 5’UTR transcribed from LINE-1 was inserted into the first intron of *ASIP*, leading to a lack of pigment in the skin and hair of white buffalo [23] (Fig. 4d). A similar LINE-1 insertion is also found in the *ASIP* gene of cattle, indicating the convergent and universal insertion of TE in different livestock and poultry species. Therefore, it is necessary to construct a global view of TE composition and evolutionary conservation to improve our comprehensive understanding of TE dynamics and their roles in livestock and poultry genomes.

### Bioinformatics strategies and methods for studying TE in livestock and poultry

**Fig. 5:**
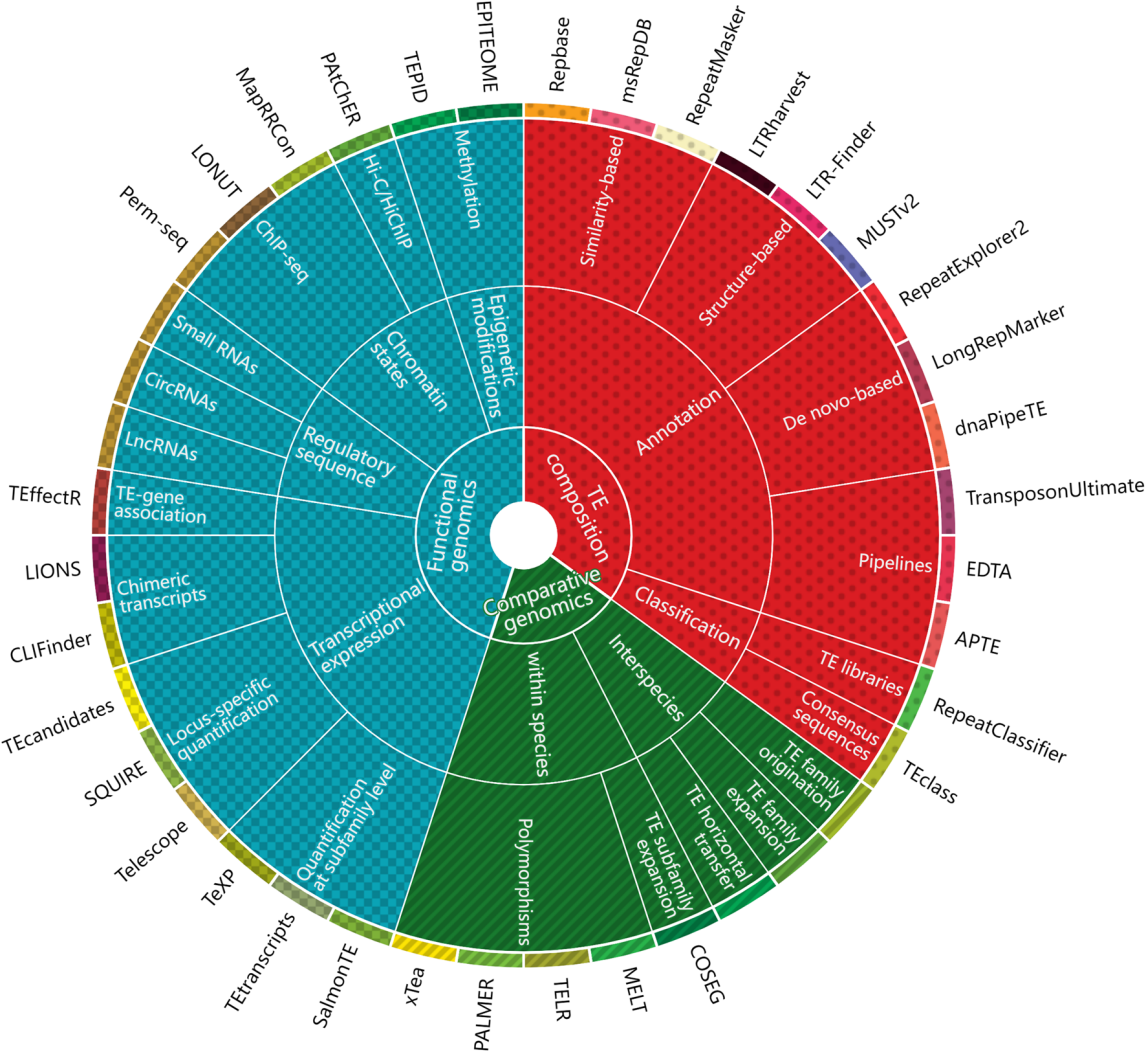
Schematic illustration of available TE research areas, strategy, method, and tools. The pie chart represents research areas, strategies, methods, and tools from the inside out. TE research areas include three aspects: TE composition (red), comparative genomics (green), and functional genomics (blue)

In recent years, a growing number of standardized methods and tools have been developed to meet the application requirements of TE in various fields of genetics, genomics, and systems biology [90]. Here, we review the representative strategies and methods (including 2 to 3 tools for each strategy) that have been used to answer key questions on the biology of TE (Fig. 5). We also discuss how these derivative tools can help elucidate the functions of TE in livestock and poultry genomes.

#### Transposable element composition

The knowledge of TE composition is the foundation of TE research, and relies mainly on TE annotation and classification systems. Existing approaches to TE annotation can be roughly classified into three categories: similarity-based, structure-based, and de novo-based strategies [91]. In similarity-based methods, genomic sequences are queried against the TE consensus sequences from known TE repositories, such as Repbase Update [24], Dfam [92], and msRepDB [93]. RepeatMasker is currently the best tool for similarity-based genome-wide TE masking [94]. Structure-based methods use the structural features (e.g., motif query) of different TE families to annotate specific TE families. For example, LTRharvest [95] and LTR-Finder [96] can be used for LTR annotation using features such as target site replication, and MUSTv2 [97] is used to identify MITE copies (DNA TEs) based on their terminal inverted repeats and direct repeats.

De novo-based methods provide consensus sequences and structural features for the first two methods, and can be used to detect unknown TE families. De novo-based strategies can also be divided according to their sequence sources, and many popular and representative tools have been developed for this method. For example, RepeatModeler2 [98] and RECON [99] use pairwise similarity or consensus seeds to cluster repetitive sequences from the assembled genomes, whereas RepeatExplorer2 [100] and dnaPipeTE [101] perform TE annotation by directly assembling and clustering (e.g., *k*-mer and self-comparison) the raw reads. Recently, LongRepMarker [102] was developed to simultaneously use genome sequences, paired-end reads, and barcode-linked reads or long reads for the comprehensive identification of TE sequences. The performance of LongRepMarker is comparable to those of traditional methods. As such, it has been used to construct the msRepDB database that covers 80,000 species and contains more complete TE families than the Repbase Update and Dfam databases [93]. Furthermore, the TransposonUltimate [103], EDTA [104], and APTE [105] pipelines have been developed to combine multiple software across the three strategies with the necessary merging and filtering steps for high-performance TE annotation.

TE consensus sequences constructed from de novo-based annotations also require further TE classification. Using search engines (e.g., RM-BLAST and cross-match) to find homologies with known TE libraries (e.g., Repbase Update) is the most common strategy for TE classification, and RepeatMasker and RepeatClassifier [98] are representative tools for this method. Another strategy to classify unknown TE consensus sequences is based on the mechanism of TE transposition, and is embodied in the TEclass tool. This tool combines support vector machines, random forests, and learning vector quantization to predict open reading frames [106]. It is worth noting that the outputs of TE annotation and classification are not ready for subsequent analysis, and the nesting structure between TE needs to be considered to avoid inaccurate understanding of transposons. A useful collection of Perl scripts (https://github.com/4ureliek) provided by Aurelie et al. can be used for the identification of nested and nesting TE. In general, TE with clear genome annotations, family classifications, structural integrity, and complexity can be used for further evolutionary and functional studies.

#### Comparative genomics

The mobility of TE is mainly reflected in comparative genome analysis within and between species. The comparison of TE composition among species reflects the different evolutionary trajectories of species. This is accompanied by the de novo origination, expansion, and reduction of TE superfamilies/families and a very small number of TE horizontal transfer events [107]. Generally, lineage-specific expansion and reduction of a TE superfamily/family can be directly identified by comparing the relationship between the changes in TE composition and speciation events [108]. In addition, Ricci et al. [109] designed two parameters—density of insertion (DI) and the relative rate of speciation (RRS)—to prove the correlation between bursts of TE activity and speciation events. In particular, the expansion of specific TE subfamilies in closely-related species (e.g., the Alu subfamilies in primate genomes [110]) can be identified using the COSEG pipeline, which uses the orthologous sequence alignment of the subfamily consensus sequence to classify the TE subfamily and construct its phylogeny.

The recent evolutionary dynamics of TE within a species are reflected in TE polymorphisms between populations or breeds and play an important role in shaping their architecture, diversity, and regulation [111]. With the increasing demand for analyzing TE polymorphisms in various studies, several software programs have been developed to detect the genotypes of polymorphic TE at the population level, even from short reads at relatively low sequencing depths. To the best of our knowledge, the MELT tool [112] performs well in detecting polymorphic TE for multiple species, and the results accurately recapitulate their known population mixing patterns. However, sequencing depth has a large impact on the detection of polymorphic TE when using short reads, and a high and uniform sequencing depth is important for unbiased population genetic analysis. Fortunately, the detection of polymorphic TE can be significantly improved with tools designed for long-read sequencing technology, which can capture the full sequence and flanking regions of inserted TE. For example, the TELR tool (https://github.com/bergmanlab/telr) can estimate the allele frequencies of TE from long-read sequence data based on local assembly methods, and the PALMER tool [113] can detect nearly twice as many L1Hs insertions as detected in previous studies using short-read sequences. Furthermore, the recently developed xTea tool [114] can use both short-read and long-read data, and has superior performance in terms of sensitivity and specificity compared to existing methods.

#### Functional genomics

Transposable elements play direct and indirect roles via various regulatory modes, making widespread contributions to gene regulatory networks associated with crucial cellular functions. The direct mode indicates instances where TE are directly involved in the formation of coding or non-coding transcripts (chimeric transcripts), and can be identified by RNA-seq and isoform sequencing (Iso-Seq). Due to their repetitive nature, TE-derived transcripts are difficult to measure using short reads from RNA-seq, and their quantification is usually limited to the subfamily level. SalmonTE (high-performance [115]), TEtranscripts [116], and TeXP [117] are representative tools for this kind of task.

More recently, several methods and tools have been developed to address the need for locus-specific quantification of TE-derived transcripts. These methods adapt different redistribution strategies for short reads and statistical methods (e.g., the EM algorithm). The typical tools include Telescope (high-performance [115]), SQUIRE [118], and TEcandidates [119]. In addition, CLIFinder [120] and LIONS [121] are specifically designed to identify fusion events or chimeric transcripts (as TE are typically used as alternative promoters) by combining split reading and paired-end algorithms. The TEffectR tool [122] was developed to directly identify the *cis*-regulatory effects of TE, and it statistically associates TE transcription and nearby gene expression based on a linear regression model. Compared with the short reads obtained from RNA-seq, the long reads obtained from Iso-Seq can dramatically reduce the proportion of ambiguously mapped reads. It helps capture complete transcripts and ensures the accurate structure of TE in chimeric transcripts, but it also poses certain limitations in terms of accurate quantification (including relatively small sample size and library size). Therefore, a combination of Iso-Seq and RNA-seq is a better strategy that greatly improves TE expression at locus-specific levels.

The indirect mode by which TE affect gene regulatory networks is mainly through contributing *cis*-regulatory sequences and generating various chromatin states (active/inactive). In addition to their above-mentioned role as *cis*-regulatory elements as part of lncRNA (via chimeric transcripts), TE can also be involved in the formation of small RNA (sRNA) and circular RNA (circRNA). sRNA can be derived from TE-expressed chimeric transcripts (i.e., TE-derived sRNA, including piRNA, siRNA, and miRNA). And they play a crucial role in promoting TE silencing (piRNA and siRNA). The formation of exonic circRNA (exon circularization) relies on the complementary sequences from the flanking introns, for which TE can be a potential source [123]. To the best of our knowledge, there are no specific computational tools that can directly combine sRNA/circRNA and TE. However, it is possible to obtain TE annotations (e.g., using RepeatMasker) and sRNA/CircRNA sets (e.g., using miRDeep2 [124]/CIRCexplorer2 [125]) separately and then establish their co-locations or overlapping relationships (e.g., using Bedtools [126]).

Chromatin states of the TE-derived regulatory elements—including enhancers, promoters, silencers, repressive elements, and transcription factors—are typically derived from chromatin immunoprecipitation followed by high-throughput sequencing (ChIP-seq) assays of histone modifications. As in other cases, ambiguously mapped reads caused by repetitive sequences are the main analytical challenge. The current strategy is to use unique reads or to apply various tools (e.g., Perm-seq [127], LONUT [128], and MapRRCon [129]) to redistribute the multi-mapped reads, which helps achieve higher specificity and resolution for ChIP-seq assays. Recently, a novel strategy was proposed through the combination of Hi-C/HiChIP (3D folding of chromatin) and the PAtChER tool, which can accurately measure TE-derived gene regulatory elements at a locus-specific level [130].

Transposable element activities result in diverse epigenetic modifications, and induced changes in the epigenetic landscape also affect nearby functional elements that can be epigenetically regulated. The sequences from most TE families are methylated in most tissues and organs over the long term, except at the embryonic stage. Enrichment-based methods (e.g., MeDIP-seq and MRE-seq) and bisulfite-based sequencing [e.g., whole genome bisulfite sequencing (WGBS), reduced representation bisulfite sequencing (RRBS), and methylated-DNA immunoprecipitation sequencing (MethylC-seq)] are the most commonly used strategies for estimating DNA methylation levels and any subset of the genome occupied by TE can be directly assessed for DNA methylation by them. Several tools, such as TEPID [111] and EPITEOME [131], also consider the probability of multi-mapping reads. This improves the detection of TE methylation levels by analyzing split reads that span connections between TE and uniquely mappable genomic regions.

### Transposable elements in the context of complex traits and animal breeding

**Fig. 6:**
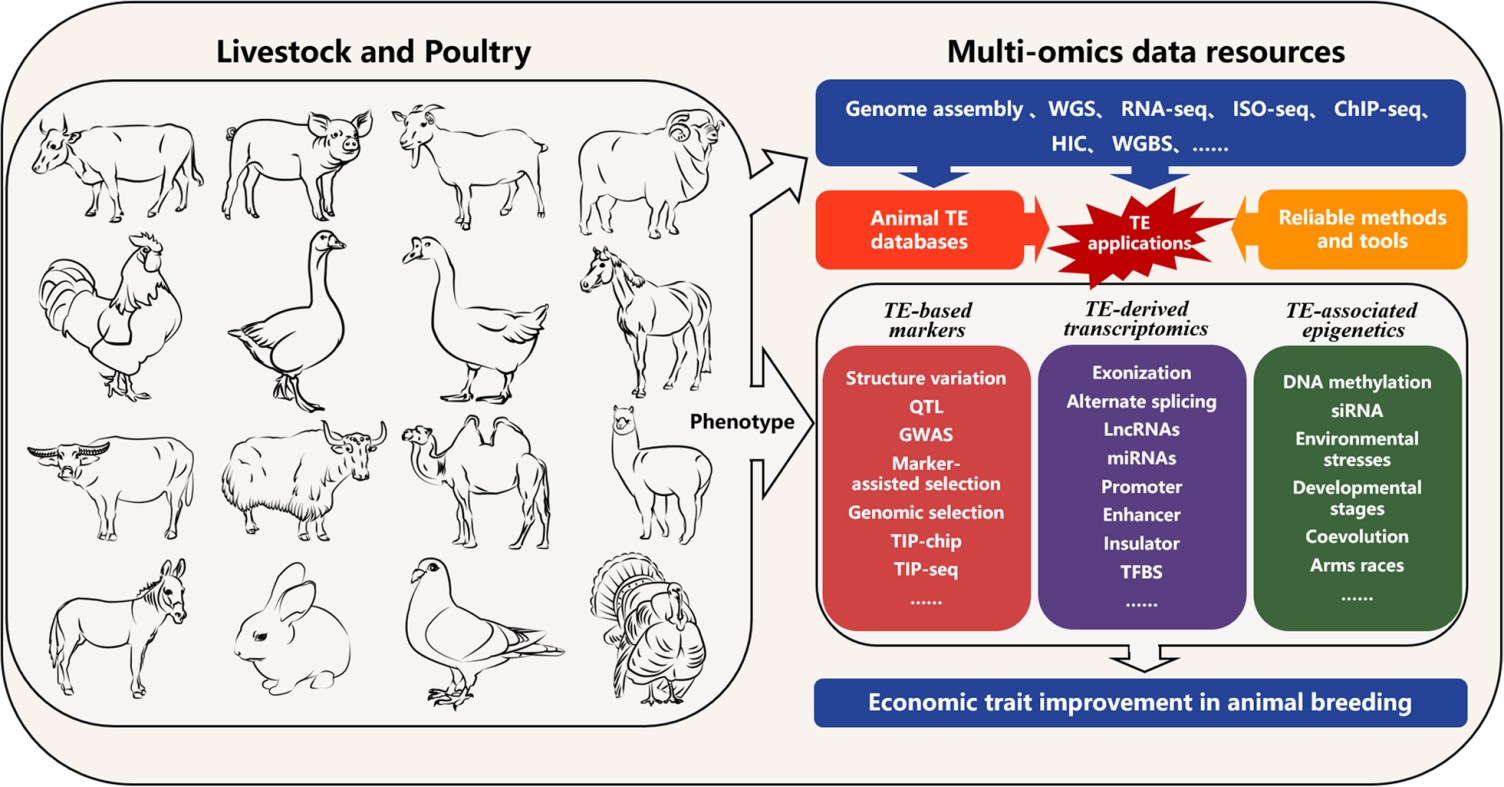
Potential applications whereby TE contribute to complex traits and animal breeding. Animal breeding can be improved through the use of TE by combining multiple omics data resources, animal TE databases, robust methods, and tools. This can be achieved through three application aspects of TE: TE-based markers, TE-derived transcriptomics, and TE-related epigenetics

In view of the current lack of knowledge regarding the role of TE in complex traits and the breeding in livestock and poultry, we summarize the major aspects and feasible strategies for TE applications in humans and plants. We provide a potential reference for the applications of TE in the field of livestock and poultry in the future (Fig. 6).

#### Development and application of TE-based molecular markers

Genetic diversity is a key basis for analyzing the economic traits of livestock and poultry and is an important premise for promoting the development of the livestock and poultry breeding industries. Therefore, it is critical to develop a comprehensive understanding of livestock population structures and lineages of genetic diversity in order to effectively use them for animal farming practices. Molecular markers are primarily based on DNA sequence variability and play an important role in basic genetic research (e.g., for constructing genetic maps and mapping quantitative trait loci) and breeding applications (e.g., marker-assisted selection and genomic selection). Transposable elements occupy nearly one-third of the livestock genome and approximately one-tenth of the poultry genome. Moreover, parts of the TE families are currently active and polymorphic, resulting in a large number of intraspecific SV. These TE-derived SV have been used to elucidate or refine the genetic relationships between breeds within a species [132].

At present, molecular markers (represented by SNPs mainly) have been widely used to study population genetic structures, germplasm resources, and DNA fingerprinting. However, there are still some limitations in the interpretation of phenotypic variance through SNPs. Studies have shown that although subsets of SV are unrelated to SNPs (i.e., no significant linkage disequilibrium) [133], SV can cause larger changes in genome structure than SNPs, may have greater functional impacts, and are more likely to be true causal variants [134]. In particular, TE-derived SV are more likely to be formed as a result of TE insertions than deletions [135]. These findings indicate that TE are informative, traceable, and can be used as reliable genetic markers.

In recent years, TE-based molecular markers have been applied in humans and in the agricultural industry with promising results. Several studies have reported a significant association between TE-associated SV and the underlying causes of cancer and genetic disorders [136, 137]. Molecular markers based on highly polymorphic TE have been used to study genetic diversity and create genetic linkage maps, making them suitable for cultivar identification and marker-assisted selection (MAS)-based breeding programs in wild and cultivated barley [138]. Genome-wide association studies in tomatoes have identified at least 40 polymorphic TE associated with extreme variations in major agronomic traits or secondary metabolites [19]. Specific agronomic traits, such as plant height and ear length traits, have been associated with allelic TE-based markers in rice [139]. Thus, the construction of TE-based molecular markers is feasible and can compensate for the limitations of other molecular markers to a certain extent. With the development of genomics, genome assembly, and sequencing technologies, it is possible to ensure the accuracy, sensitivity, and comprehensiveness of polymorphic TE detection across livestock and poultry breeds by integrating reliable tools (e.g., MELT) and newly developed algorithms (e.g., PALMER and xTea). Therefore, taking cues from the current applications of TE in humans, it is possible for the agricultural sector to construct TE-based genotyping chips to detect polymorphic TE in livestock and poultry at the population level.

In general, three main steps are required to perform large-scale population screening for TE polymorphisms, rapidly and efficiently. The first step involves producing polymorphic TE datasets for each species, which can be obtained using multiple assembled genomes, long-read sequencing (PacBio and Nanopore), and short-read sequencing [140]. Short-read sequencing only shows good performance for detecting deletion-type TE (relative to the reference genome) because of its limitations in obtaining inserted TE sequences [141]. In contrast, assembled genomes and long sequences are the best options for capturing the precise sequence composition of polymorphic TE [142, 143]. The next step is to design specific locus-flanking sequences for all or candidate polymorphic TE; these unique sequence tags serve as the basis for identifying the location of polymorphic TE in the genome. Finally, population-level genotyping can be accomplished based on these sequence tags using sequence-based assays. For example, high-intensity unique sequence tags can be designed to probe TE using the microarray method (TIP-chip) [144], or TE can be PCR-amplified and detected using high-throughput sequencing (TIP-seq) [145]. At present, this step has only been accomplished in a few livestock and poultry breeds, and most of these studies have been limited to detecting polymorphic TE based on short-read sequencing [41]. Therefore, we believe that more attention needs to be paid to TE polymorphisms and that it is necessary to develop and apply TE-based molecular markers to livestock and poultry genomes.

#### TE-derived transcriptomes and their roles in regulatory networks

Transposable elements can affect the transcriptome in different ways [135]. The most direct way is through TE-induced changes in the sequence of the protein-coding gene. For example, the insertion of human TE into the exon of a coding gene can disrupt the original sequence structure and generate “exonization” events that are one of the main causes of human diseases [146]. Most TE exonizations result in alternate splicing of internal exons, eventually leading to new alternative splicing events [147]. However, because of the limited number of existing studies on livestock and poultry genomes, only a few exonization events (e.g., LINE2 exonization in the horse *MSTN* gene) have been reported to date, and most TE insertions occur in the untranslated region of the coding gene (e.g., the first exon). Therefore, it is worthwhile to consider the effect of TE on exonization and alternative splicing events following conventional RNA-seq analysis of livestock and poultry. In this regard, Iso-Seq is a good option for improving the identification of novel TE-derived transcripts and providing locus-specific TE expression levels [148].

Transposable elements can also serve as an important source of functional lncRNA and small non-coding RNA (miRNA and siRNA) [149, 150]. These TE-derived non-coding RNA are closely associated with specific stress conditions [151] or developmental stages [152], and are currently less studied in livestock and poultry. However, these offer enormous research potential owing to their roles in functional genomics. TE-derived small RNA can influence the *trans*-regulation of protein-coding gene activity at the transcriptional and post-transcriptional levels through sequence complementarity [152]. Based on the association of small RNA with specific TE families, the evolutionary history and conservation of TE families can be effectively used to better understand the evolutionary and functional properties of small RNA in livestock and poultry. In the past five years, transcriptomic analysis has greatly expanded the catalog of lncRNA in livestock and poultry [153]. Thus, it has been adopted as a routine approach for profiling global transcriptome changes across tissues, developmental stages, breeds, and environmental stresses [154]. However, the role of TE in lncRNA has not been fully investigated, and the biological functions of TE-derived lncRNA have been underestimated.

In addition to forming transcripts, TE can indirectly influence gene regulatory networks as *cis*-regulatory elements [155]. Studies have shown that chromatin accessibility and histone modification patterns are highly correlated with the presence and family of TE. Even specific TE families can introduce new enhancers or promoters that comprise functional TFBS, which can spread throughout the genome with TE amplification [7, 156, 157]. The expansion of TE-derived TFBS can help elucidate the species-specific functions of transcription factors [155, 158], which may be an important driving force for shaping the regulatory networks of livestock and poultry.

#### TE-associated epigenetics

Because TE mobilization can lead to genomic instability, it is strongly inhibited by epigenetic silencing mechanisms [159]. This TE silencing mechanism may affect the transcriptional activity of adjacent genes by modulating the epigenomic profile of their close regions or by altering the activity of their neighboring regulatory elements [160]. In general, the epigenetic silencing of TE is relatively stable in most somatic cells, but highly active in specific biological processes (e.g., during reprogramming in germ cells and pre-implantation embryos) and environmental stresses [161]. The activation of epigenetically silenced TE has been found to be a novel mechanism of oncogene activation known as TE onco-exaptation events [162]. The LINE1 element—which controls leaf senescence and allows plants to adapt to a local climate by regulating the expression of the *pheophytinase* (*PPH*) gene [163]—was found to be differentially methylated in *Arabidopsis* accessions. Therefore, changes in TE-related epigenetic signatures are functional and are worthy of attention in studies on livestock and poultry.

At present, there are some limitations in evaluating the methylation level of TE using the unique mapping reads obtained from NGS-based sequencing (e.g., WGBS and RRBS). In this regard, the Oxford Nanopore long-read sequencing technology offers an excellent system for the simultaneous identification of TE polymorphisms and methylation levels in the TE body [164, 165]. Standard DNA methylation-calling tools and workflows for nanopore sequencing have been designed for modified base detection at the genome scale, and can serve as the basis for relevant studies in livestock and poultry species [166]. Using these techniques, we can compare the methylation of different animal breeds across geographical distributions or explore how TE affect the changes in methylation at different developmental stages.

Another point that deserves special attention relates to “coevolution” or “arms races” between TE and their livestock and poultry hosts. Although silencing mechanisms can prevent TE amplification, TE can evade this host machinery through recurrent evolutionary innovations [167]. This complex relationship facilitates not only the expansion of TE families but also the functional evolution of the host organism. In particular, ERV are a typical example that has been shown to be indispensable in livestock and poultry, as described above. However, it is necessary to perform a series of studies that integrate the domestication and epigenetic components of livestock and poultry and compare their transcriptional activities for lineage-specific ERV.

## Conclusions

Transposable elements are important components of livestock and poultry genomes, representing approximately 26.1 to 42.9% of the entire genome. The mobilization, transcriptional regulation, and silencing mechanisms of TE have substantial impacts on the variability of the genome, transcriptome, and epigenome in livestock and poultry. Furthermore, TE have the potential to contribute to phenotypic variation in complex traits. By investigating the effects of TE activity on host fitness in livestock and poultry, researchers could identify areas where research is needed to improve animal health and productivity. However, current research on TE in livestock and poultry is still in its infancy and not as extensive as that conducted on humans and other model animals, such as mice and fruit flies. Although studies on TE in livestock, such as pigs and chickens, have been gradually increasing, they are limited to specific research directions, and the number of studies on these species is very small (less than 20). Specifically, research on TE silencing mechanisms and epigenetic regulation, as well as the relationship between polymorphic TE and actual/molecular phenotypes, is limited. This is in stark contrast to the rapid development of livestock functional genomics and the accumulation of multi-omics data. To improve research on TE in animal breeding and research, it is important to establish standardized bioinformatic tools/methods for data collection, analysis, and reporting. In addition, data sharing between researchers and institutions can help accelerate progress in TE studies. Exactly as the recent developments in the farm animal pan-genomes, functional annotation of animal genomes (FAANG), and farm animal genotype-tissue expression (FarmGTEx) projects provide excellent opportunities for studying TE. Although various challenges still exist, we believe that with the accumulation of multi-omics data in recent years, it is a good time for researchers to start using transposons as a routine analytical tool in livestock and poultry research.

## Data Availability

Not applicable.
